# Automated classification of shoulder radiology focusing on cuff tear arthropathy and glenoid erosion using AI

**DOI:** 10.1186/s12891-026-09603-5

**Published:** 2026-02-25

**Authors:** Michael Axenhus, Martin Magnéli, Jacob Gislén, Johan Fagrell, Petter Ling, Yilmaz Demir, Erica Domeij Arverud, Kristofer Hallberg, Björn Salomonsson, Max Gordon

**Affiliations:** Department of Clinical Sciences at Danderyd Hospital, Division of Orthopaedics, Karolinska Institutet, Stockholm, Sweden

**Keywords:** Artificial intelligence, Automated classification, Cuff tear arthropathy, Glenoid erosion, Shoulder radiology

## Abstract

**Background:**

Recent advancements in the field artificial intelligence (AI), particularly in the architecture of convolutional neural network (CNN) architecture, have revolutionized medical imaging by enabling accurate image recognition. However, the application of AI in identifying degenerative musculoskeletal disorders, specifically on plain radiographs, is still poorly explored. The aim of this study is to classify cuff tear arthropathy (CTA) and glenoid erosion using AI on plain shoulder radiographs, using the Hamada and Favard classification systems.

**Methods:**

We used a publicly available CNN trained for image recognition and trained it using a diverse dataset of 6733 shoulder and clavicle X-ray images covering various clinical conditions. The performance of the network was evaluated in detail on a validation set of 561 images. Metrics such as sensitivity, specificity, Youden’s index, and Area Under Curve (AUC) in the receiver operating characteristics curve analysis, were used for evaluation. AUC was the primary measure of accuracy.

**Results:**

The network showed exceptional performance in identifying Hamada grades 3 and 4, achieving AUCs of 0.95, 95% CI [0.91–0.98] for both categories. While performance was slightly lower for Hamada grades 0–2 and glenoid erosion, with AUCs ranging from 0.81 to 0.91, it still demonstrated considerable accuracy. Similar results were obtained for Favard although we could not validate the network for more advanced stages due to lack of data.

**Conclusions:**

Our study demonstrates the network’s robust capability to identify CTA on plain radiographs, comparable to earlier studies focused on osteoarthritis. Notably, the network excelled in later disease stages characterized by pronounced pathology. The ability to achieve such performance with a heterogeneous dataset bodes well for the real-world implementation of AI technology. However, it is crucial to acknowledge the potential influence of using a validation set instead of a dedicated test set, warranting further investigation.

## Background

Cuff tear arthropathy (CTA) is an independent shoulder pathology associated with chronic rotator cuff deficiency. It was first documented by Neer et al. [1] and subsequently studied by other researchers [2, 3]. Despite its clinical significance, CTA has not been officially recognized as a separate entity in diagnostic classifications. Nevertheless, there is agreement of the defining features: a chronic rotator cuff tear, upward displacement of the humerus in the glenoid fossa leading to progressive wear on the superior aspect of the glenoid, and morphological changes such as acetabularization of the underside of the acromion and femoralization of the head of the humerus [3, 4]. Radiologically, this disease shows a similar picture to glenohumeral osteoarthritis (OA) characterized by an ascending humeral head and erosion of the glenoid.

In the context of shoulder pathology, rotator cuff disorders are widespread and affect up to 70% of patients with shoulder pain, ranging from tendonitis to complete cuff tears [5]. Epidemiological studies, both in cadavers and living subjects, show a consistent increase in prevalence with each decade of life, reaching up to 80% in the eighth decade [6, 7]. Damage to the rotator cuff not only alters the biomechanics of shoulder movement but also increases the risk of OA. It is estimated that about one third of patients with cuff tears are symptomatic and some progress to develop CTA [1, 8]. As CTA progress, significant bony degeneration occurs, leading to end-stage complications such as humeral head collapse, known as avascular necrosis (AVN) [9]. At this point, surgical intervention like hemiarthroplasty become a viable option [2]. CTA, which is often due to a rotator cuff tear, represent a difficult treatment dilemma that emphasize the importance of considering early surgical repair, especially for full-thickness tears [1].

The field of Artificial intelligence (AI)-assisted analysis has so far mainly focused on cross-sectional radiology, with studies primarily concentrating on non-orthopedic imaging such as mammography and thoracic radiography [10]. There is particularly little research on shoulder radiography in this context, except for a few studies focusing on fractures [11].

Several classification schemes have been proposed to describe CTA, the best known of which are named after Hamada, Seebauer, and Favard [3, 9, 12]. These schemes aim to delineate degenerative changes of the glenoid, humeral head, and acromioclavicular arch. In particular, migration of the humerus is a key feature in the Hamada and Seebauer classifications.

To our knowledge, no previous study has used an artificial neuronal network (ANN) to classify CTA or other degenerative disease of the shoulder on plain radiographs. Similar studies have been performed on degenerative disorders of the knee and hip and were successful [13, 14]. We hypothesize that an ANN can be used to classify CTA according to known classification schemes.

The aim of this study was to train and evaluate the performance of a CNN in classifying of CTA according to the Hamada and Favard systems on plain radiographs.

## Materials and methods

### Study design and sample

A total of 6733 plain X-ray examinations were extracted from the Picture Archiving and Communication System (PACS) at Danderyd University Hospital, Sweden. All examinations were performed between 2002 and 2016 for clinical purposes, and each examination consisted of 2–7 radiographs. Examinations were anonymized during the extraction process and were void of all patient data. Ethical permit was granted by the Stockholm Ethical Review Board, Sweden. Dnr: 2014/453 − 31/3. The Stockholm Ethical Review Board waived the need for informed consent for this study. Radiologists’ reports were included for each examination when available.

### Datasets

**Fig. 1:**
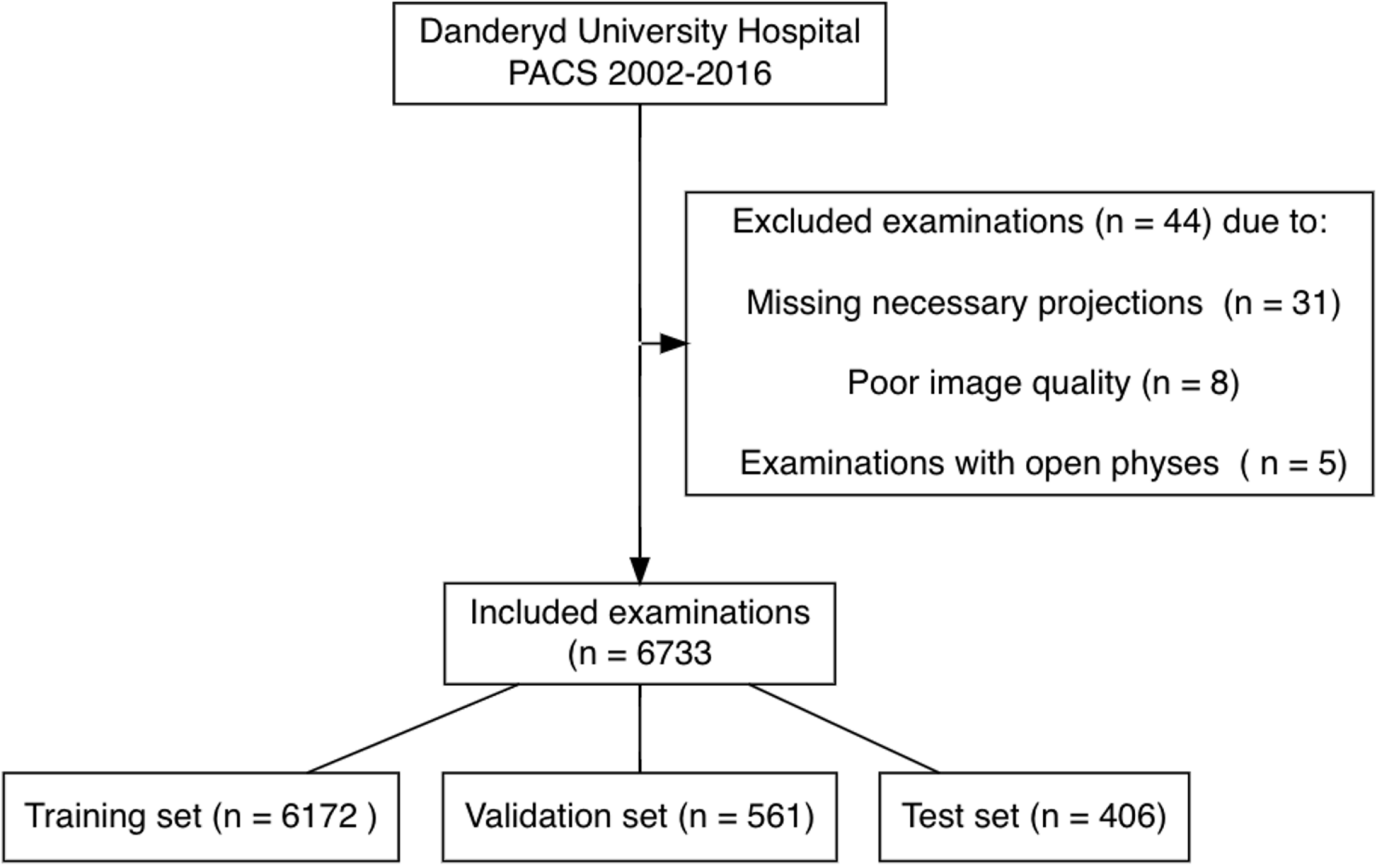
Dataset flow chart with training, validation and test set

The study sample (*n* = 7139) dataset was divided into three subsets for training (*n* = 6172), validation (*n* = 561), and testing (*n* = 406) **(**Fig. 1**).** There was no patient overlap. Radiographic examinations of the shoulder were chosen based on radiologist reports, without specific consideration of projection types. Classes with poor performance in initial network classification were pinpointed for enhancement. Active learning was employed to boost performance, involving the inclusion of more examinations from the specific fracture class. The PACS database was scrutinized for radiologist reports hinting at the selected fracture types, introducing a potential selection bias. However, this was deemed acceptable to enhance prediction precision across all classes.

### Labelling of radiographic examinations

The extracted radiographic examinations were uploaded to an in-house developed online labelling platform and were labelled with respect to CTA and glenoid erosion. The training and validation datasets were labelled by three 4th-year medical students. Radiologist’s reports were attached to each examination when available, complementing the students in visual assessment. The labels were considered ground truth. Particularly ambiguous or difficult cases were revisited and re-audited by the medical students, often with senior surgeon supervision. The test set was labelled by four senior shoulder surgeons.

### Diagnosis and Classification

**Fig. 2:**
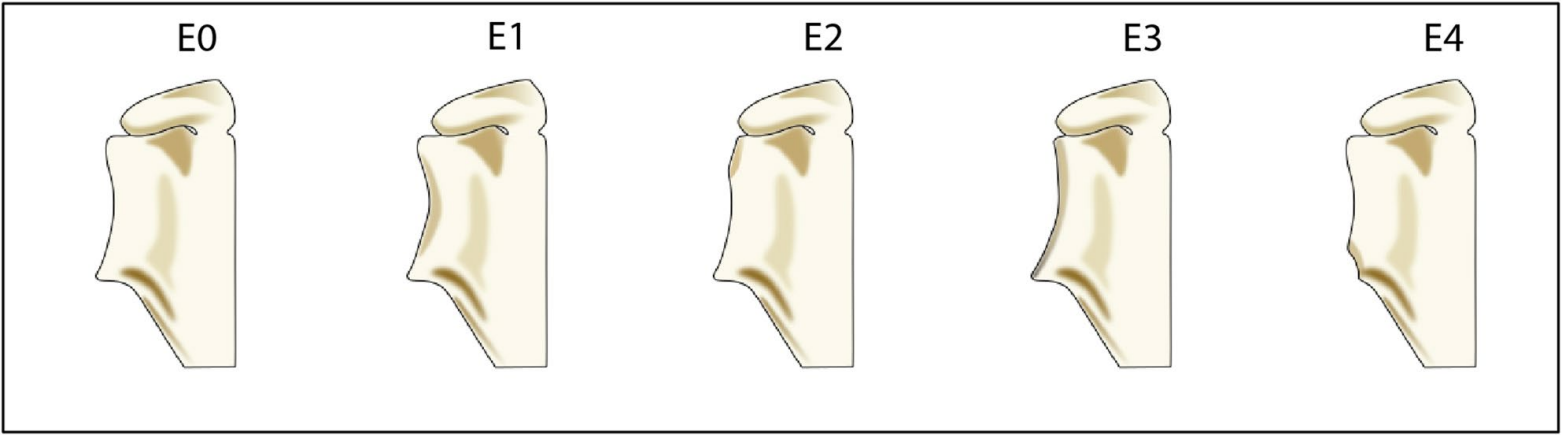
Favard classification for glenoid erosion. Each grade shows the glenoid with different patterns of erosion. E0: superior humeral migration with no glenoid erosion. E1: Concentric glenoid erosion. E2: Superior glenoid erosion. E3: Superior glenoid erosion extending to the bottom of the glenoid. E4: Inferior glenoid erosion

**Fig. 3:**
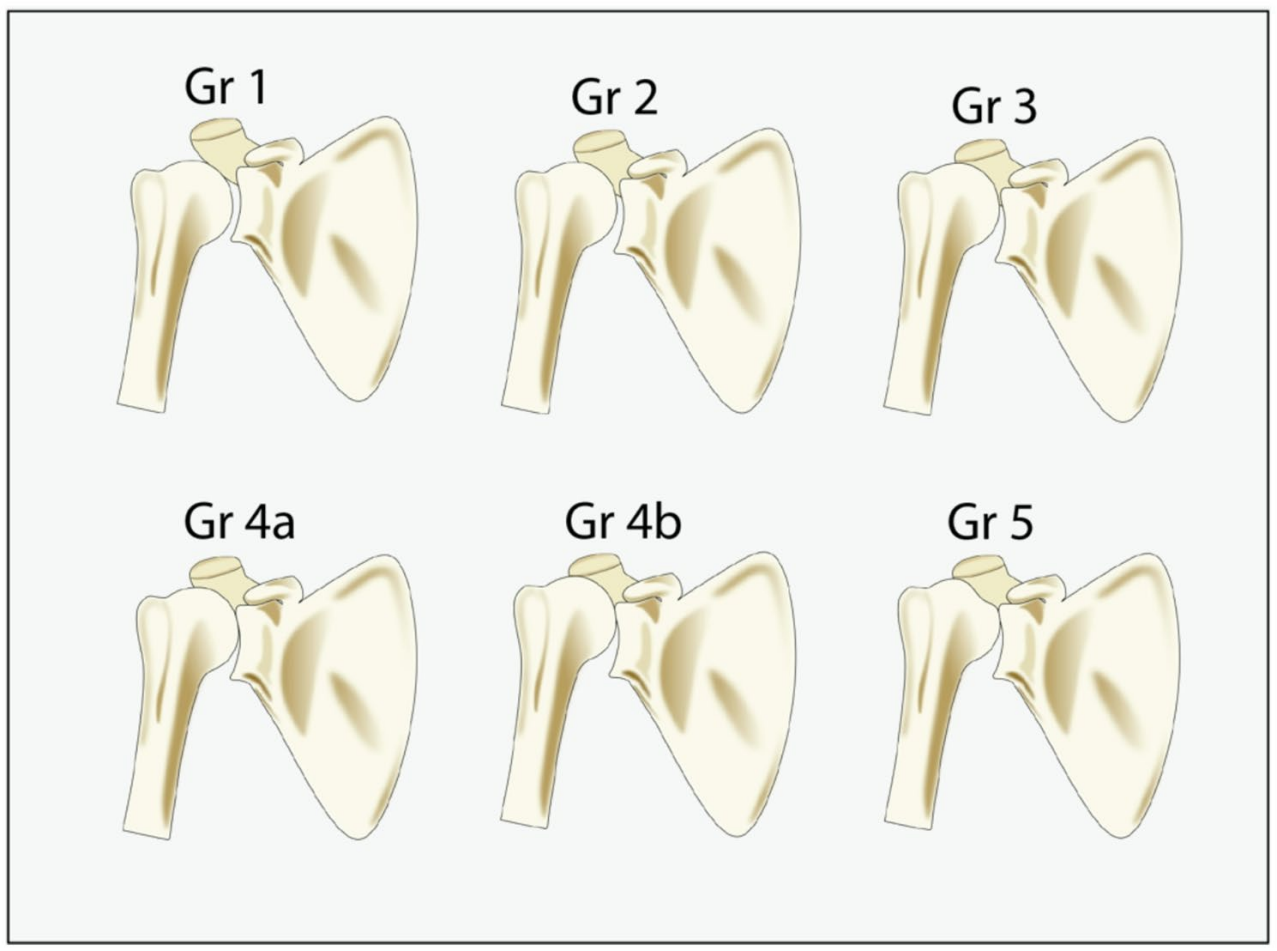
Hamada classification for CTA. *Grade 1*: Superior humeral migration with acromio-humeral interval greater than 6 mm. *Grade 2*: AHI <5 mm. *Grade 3*: Grade 2 changes with acetabularization. *Grade 4 A*: Superior humeral migration with glenohumeral narrowing and sclerosis without acetabularization. *Grade 4B*: Superior humeral migration with glenohumeral narrowing and sclerosis with acetabularization. *Grade 5*: Humeral head collapse, avascular necrosis

Glenoid erosion was labelled according to the Favard system (Fig. 2) [15]. CTA was assessed using the Hamada Classification (Fig. 3) [16]. As the Hamada system aims to classify radiographs of patients with a demonstrably damaged cuff, we have labelled healthy shoulders Hamada 0. The description of Hamada 1 is an AHI of more than 6 mm, all healthy shoulders could therefore be labelled Hamada 1. In this study we did not know if the patient had been diagnosed with a cuff tear, so Hamada 1 was used in cases with apparent superior humeral migration with AHI no lower than 6 mm. When labelling the training set, Favard was labelled on images with superior humeral migration or, in the case of grade E4, glenoid erosion in the anteroinferior pole. Labels were applied in a hierarchical manner, with the option of not assigning groups and/or subgroups that could not be determined by the observer. The outcome variable was classification according to the Favard classification system and the Hamada classification system.

### Review and Labeling Process

The training and validation datasets were labeled by three 4th-year medical students from Karolinska Institutet, Stockholm, Sweden. The test set was labeled by four senior consultant orthopedic surgeons specialized in shoulder surgery. The labels served as a ground truth. The examinations were labeled on a custom-built online platform, where tools to label plain radiographic images based on the CTA classification according to Hamada with Favard classifications used to classify glenoid erosion. All images with degenerative labels were additionally reviewed by all three reviewers, to reduce potential label noise caused by interobserver inconsistency.

### Labeling

As the network was trained, it gradually learned to predict labels for each examination during subsequent labeling. The network’s predictions were incorporated into the online labeling platform and were presented to the human observers as a degree of network certainty ranging between < 50% certainty, 50–70% certainty, or > 70% certainty of selected labels. The human observers had the choice of keeping the network-predicted labels for the particular examination or changing the labels based on their own assessment of the study.

The following conditions excluded Hamada and Favard classification:

1. Examinations including fractures of the proximal humerus or other deformities to the glenohumeral joint.
2. Examinations with projections that did not properly enable classification.
3. Examinations with any form of glenohumeral implant.

### Training the CNN and evaluating CNN classification performance

The network used was a modified ResNet type [17], consisting of a total of 35 layers with batch normalization for each convolution layer and adaptive max pool. Stochastic gradient descent was employed to train the network. The 6,733 student-labelled examinations were used as training examples in CNN training and were considered ground truth in this setting. The labeled images were scaled down, resulting in 256 × 256 pixels to fit the structure of the network, with no zoom applied. Each image was subjected 80 iterations during training. To increase the robustness of the training, each image was also rotated, flipped, and randomly cropped. During the training, the model was evaluated using the validation dataset, comprising 562 examinations. The final, adjusted model was evaluated using the test dataset, comprising 406 examinations.

### Statistics

Receiver operating characteristics (ROC) curve, area under the ROC curve (AUC), sensitivity, specificity, and Youden index (J) were calculated to evaluate the performance of our network for each outcome. A ROC curve is obtained by plotting sensitivity against 1 minus specificity and serves as a probability curve at varied thresholds [18]. AUC shows the ability of the model to discriminate between studies with disease and those without disease [18] and served as the primary outcome measure. AUC is a value between 0 and 1. For this study, we defined AUC between 0.7 and 0.8 as “acceptable,” AUC between 0.8 and 0.9 as “excellent,” and AUC 0.9 or higher as “outstanding,” based on an article by Mandrekar on diagnostic test assessment [19]. An AUC of 1 represents perfect diagnostic accuracy, whereas an AUC of 0.5 indicates a random classifier [18]. AUC was presented as an absolute value with a 95% confidence interval (CI). J summarizes the maximum potential of a diagnostic test and is defined as sensitivity plus specificity minus 1 [20]. J is a value between 0 and 1. Statistical analysis was performed using a publicly available version of R with MLmetrics packages and OptimalCutpoints for sensitivity, specificity, and J.

### Ethical considerations

An ethical permit has been granted by the Ethical Review Board in Stockholm, Sweden (Dnr 2014/453 − 31/3).

## Results

Cuff tear arthropathy.

**Fig. 4:**
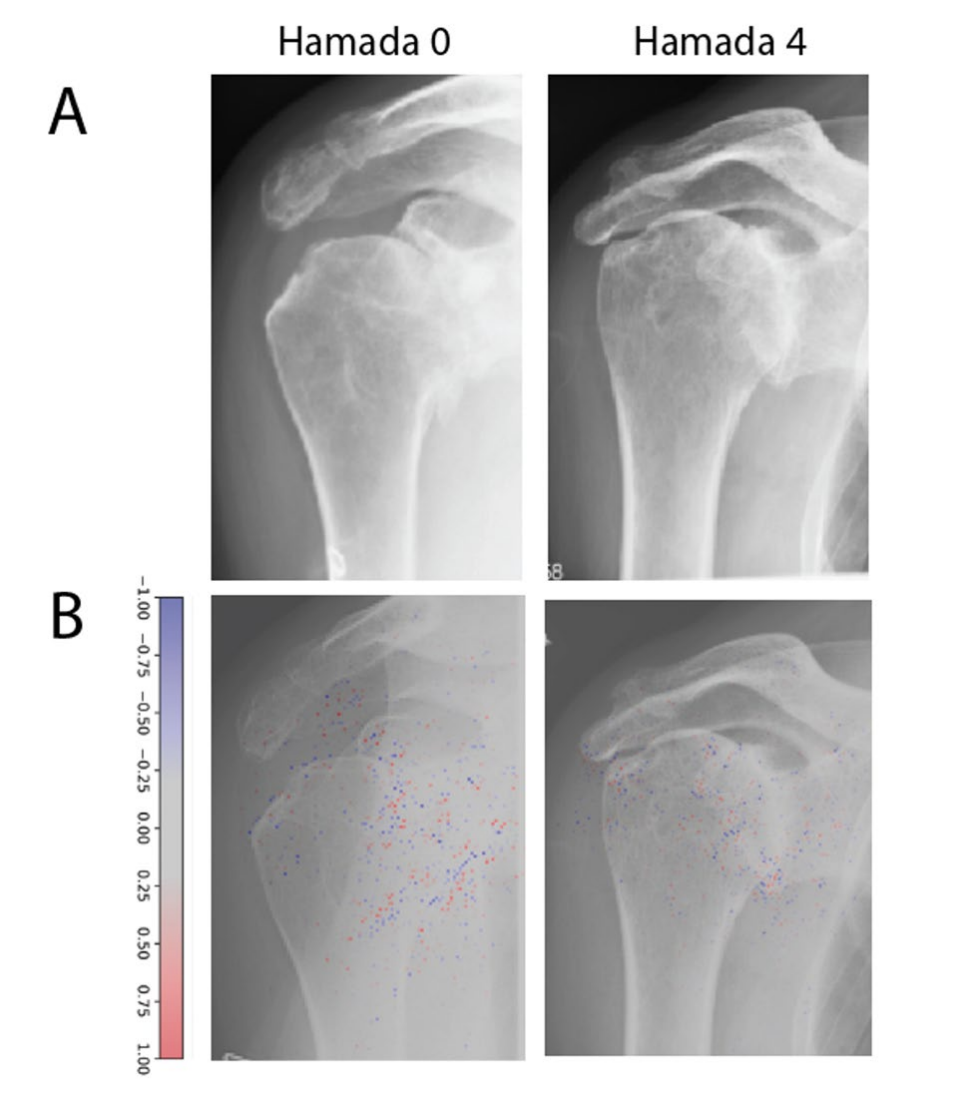
Examples of Hamada grades (**A**). AI gradient is provided (**B**)

5381 (80%) of 6733 examinations were labelled with a Hamada grade (Fig. 4). The most frequent grade was 0 (*n* = 4524), followed by 1 (*n* = 486) (Table 1). The frequency fell steadily in rising grades, with 56 cases being classed grade 5. The Favard system showed a similar pattern with 413 cases showing no glenoid erosion despite humeral ascension (E0). The most common type of glenoid erosion was E1, or concentric erosion.

**Table 1:** Distribution of Hamada and Favard grades in training and validation sets. n = number of cases (%) = percentage of total

	Training set		Validation set	
	Yes	No	Yes	No
*n* (%)	*n* (%)	*n* (%)	*n* (%)	
**Hamada**				
0	4524 (73.3%)	1648 (26.7%)	373 (66.5%)	188 (33.5%)
1	486 (7.9%)	5686 (92.1%)	27 (4.8%)	534 (95.2%)
2	119 (1.9%)	6053 (98.1%)	11 (2.0%)	550 (98.0%)
3	75 (1.2%)	6097 (98.8%)	11 (2.0%)	550 (98.0%)
4	166 (2.7%)	6006 (97.3%)	17 (3.0%)	544 (97.0%)
4a	84 (1.4%)	6088 (98.6%)	8 (1.4%)	553 (98.6%)
4b	81 (1.3%)	6091 (98.7%)	9 (1.6%)	552 (98.4%)
5	56 (0.9%)	5064 (82.0%)	8 (1.4%)	553 (98.6%)
**Favard**				
E0	413 (6.7%)	5759 (93.3%)	62 (11.1%)	499 (88.9%)
E1	130 (2.1%)	6042 (97.9%)	20 (3.6%)	541 (96.4%)
E2	33 (0.5%)	6139 (99.5%)	3 (0.5%)	558 (99.5%)
E3	20 (0.3%)	6152 (99.7%)	1 (0.2%)	560 (99.8%)
E4	18 (0.3%)	6154 (99.7%)	0 (0.0%)	561 (100.0%)

### Alternative pathology

The largest groups of alternative pathologies in the training set were fractures (28.8%), tendon calcification (13%) and shoulder dislocation (9.9%) (Table 2). The most common fractures were fractures of the clavicle and proximal humerus.

**Table 2:** Commonly appearing alternate pathology in the training and validation sets. n = number of cases (%) = percentage of total. NA = not applicable

	Training set			Validation set		
	Yes	Maybe	No	Yes	Maybe	No
*n* (%)	*n* (%)	*n* (%)	*n* (%)	*n* (%)	*n* (%)	
**Fractures**						
All	1778 (28.8)	0 (0.0)	4394 (71.2)	235 (41.9)	0 (0.0)	326 (58.1)
Clavicle	742 (12.0)	7 (0.1)	5423 (87.9)	87 (15.5)	0 (0.0)	474 (84.5)
Humerus	806 (13.1)	19 (0.3)	5347 (86.6)	123 (21.9)	1 (0.2)	437 (77.9)
Humerus, diaphysis	218 (3.5)	0 (0.0)	5954 (96.5)	39 (7.0)	0 (0.0)	522 (93.0)
Humerus, distal end segment	23 (0.4)	0 (0.0)	6149 (99.6)	1 (0.2)	0 (0.0)	560 (99.8)
Humerus, proximal segment	573 (9.3)	18 (0.3)	5581 (90.4)	84 (15.0)	1 (0.2)	476 (84.8)
Scapula	244 (4.0)	27 (0.4)	5901 (95.6)	32 (5.7)	4 (0.7)	525 (93.6)
**Others**						
Dislocation humeroscapular	609 (9.9)	NA	5563 (90.1)	70 (12.5)	NA	491 (87.5)
Calcification	804 (13.0)	NA	4316 (69.9)	47 (8.4%)	NA	514 (91.6%)
Implants	133 (2.2)	NA	6039 (97.8)	31 (5.5)	NA	530 (94.5)

Network performance.

For Hamada grade 4, the network achieved a sensitivity of 100% and specificity of 80% with an AUC of 0.95. There was more ambiguity in the lower grades, with the network performing worst in classing Hamada 0 sensitivity: 75%, specificity 78%, Youden’s 0.52 and AUC 0.81. The network was able to accurately classify concentric glenoid erosion (E1), achieving an AUC of 0.91 (Table 3).

Some grades were found to be quite rare, resulting in a very low of number of cases in a very low number of cases in the validation set. Mainly Hamada grade 5 and Favard grades E2, E3, E4, with the last two not represented in the results.

**Table 3:** Primary outcome measures on Hamada and Favard. n= number of cases, AUC = area under ROC-curve

Favard	*n*	Sensitivity	Specificity	Youden’s index	AUC (95% CI)
E0	62	79	82	0.61	0.85 (0.79 to 0.91)
E1	20	100	70	0.70	0.91 (0.86 to 0.95)
E2	3	100	53	0.53	0.75 (0.48 to 1.01)
**Hamada**					
0	373	75	78	0.52	0.81 (0.78 to 0.85)
1	27	85	77	0.62	0.85 (0.79 to 0.90)
2	11	82	86	0.68	0.84 (0.67 to 1.00)
3	11	91	93	0.84	0.95 (0.91 to 0.98)
4	17	100	80	0.80	0.95 (0.91 to 0.98)
4a	8	100	82	0.82	0.92 (0.88 to 0.97)
4b	9	100	83	0.83	0.96 (0.92 to 1.00)
0 to 1	400	72	82	0.54	0.84 (0.81 to 0.87)
1 to 3	49	86	85	0.71	0.88 (0.83 to 0.93)
5	8	88	84	0.72	0.85 (0.70 to 0.99)

Comparative examples from other representative classes showing that the network performed better on clavicle diaphysis fractures (AUC 0.98) and worse on scapular fractures (AUC 0.77) (Table 4).

**Table 4:** Outcomes for other representative classes. n = number of cases. AUC = area under ROC-curve

Fracture	*n*	Sensitivity	Specificity	Youden’s index	AUC (95% CI)
Clavicle, diaphysis	66	98	95	0.93	0.98 (0.95 to 1.01)
Humerus	124	92	92	0.84	0.97 (0.96 to 0.99)
Scapula	35	61	80	0.41	0.77 (0.69 to 0.85)

## Discussion

In this study, we systematically trained a CNN designed to detect and classify CTA on plain radiographs and thoroughly evaluated its performance. Our results show that the CNN’s exhibit significant accuracy in this complex task. In particular, the network excelled in identifying advanced stages which are characterized by substantial degeneration of bony structures. However, the classification of early stages, characterized by subtle positional changes in anatomy, was more challenging. The assessment of glenoid erosion also proved difficult, although the network demonstrated an ability to differentiate between the Favard classes that were possibly to analyze.

The network achieved an AUC of 0.95 in classing Hamada grade 3 and 4. Pooling grades 0–2 resulted in an AUC of 0.84. Results for glenoid classification were similar with AUC of 0.85 and 0.91 for grades E0 and E1, respectively. The performance level in our study mirrors previous studies on OA. Von Schacky et al. showed similar performance on detection of radiographic OA features in the hip, with an AUC between 0.86 and 0.94 for different features [13]. Thomas et al. showed network performance in grading of knee OA according to Kellgren & Lawrence (KL) with Cohen kappa value of 0.86 compared to a radiologist committee [21]. Tiulpin et al. developed a network that achieved an impressive AUC of 0.98 in determining the presence of radiographic knee OA (KL > 2) [14].

Hamada grade 0 (no changes) and grade 1 (AHI>6 mm) are differentiated by subjective migration of the humeral head. The lack of an objective measure could lead to variance between identifiers conclusions. These early stages of CTA are difficult to stage using only radiology, in lack of clinical data on function and symptoms for the diagnose. Since the position of the humerus naturally varies greatly depending on muscle tone and positioning, it is difficult to differentiate a temporary high position from humeral ascension caused by cuff pathology [22]. Hamada grade 5 is synonymous with AVN in our project, leading to images with AVN not caused by CTA to be excluded from receiving a lower Hamada grade although this case was rare in our dataset.

Erosion of the glenoid is quite difficult to assess on non-cross-sectional imaging like plain radiographs [23]. The anterior cusp of the glenoid may obscure posterior erosion and vice versa. A slightly off-axle radiographic projection may also cause the image to imitate concentric glenoid erosion. This difficulty in classification can lead to higher levels of label noise. Despite this, the network performed quite well on concentric glenoid erosion (Favard E1). The validation set did not include enough images in the other grades to judge network performance in those cases. A larger test set is required to verify our preliminary findings for Favard E1. This is an indication that a network can be trained to detect different types of glenoid erosion.

Plain radiographs of the shoulder are widely used in the emergency department, and are the first choice for imaging the extremities [24]. Our study presents a proof-of-concept for developing a system that performs accurately classify heterogenous image sets while overcoming real-world challenges of suboptimal image quality, multiple radiological projections, and multiple diagnoses in a single image series. By using a large, heterogeneous dataset, the model reflects real-world clinical variability, making it potentially valuable in routine practice. Its ability to detect subtle radiographic changes may aid in earlier diagnosis and treatment planning. This kind of versatile system holds promise for improving radiologist performance, which will be particularly impactful in cases of low radiologist availability such as in low-resource settings or during call hours.

The results of previous studies using deep learning to identify fractures and OA, and the results of our network, suggest that degenerative conditions are more challenging in the training process. It is possible that this is a reflection of human performance in labelling showing a similar pattern, especially with non-expert labelers as in our case [25]. Fractures being more binary than the continuous nature of the progress in development of OA may lead to less accurate labels and higher label noise [26]. We see also that scapular fractures that are not easily visualized on plain radiographs, often requiring computed tomography scan to improve diagnostics, proved similarly difficult for the network to detect.

To our knowledge this is the first study conducted with a dataset that has such a degree of heterogeneity and does not include information on the indication for, or question to, the radiographic examination. The study is based on an image set including large amounts of distractions, inclusion of isolated clavicle projections and image series that did not contain the joint to be classified. By contrast von Schacky et al. and Tiulpin et al. used only anteroposterior radiographs of their respective focus joint from OA datasets [13, 14]. Our study design also allowed concomitant analysis on proximal humerus fractures and glenohumeral OA in two sister studies. We believe this approach demonstrates that our method can produce a network with a broader analytical ability, capable of simultaneous radiological classification in many different systems: like recognizing a clavicle fracture and glenohumeral arthritis in the same image series. This kind of dexterity will greatly increase the utility of AI systems in clinical settings.

CTA is a radiologically complex entity with degenerative changes occurring in many different parts of the shoulder joint. Results from earlier studies show that it seems relatively easier to train networks in fracture classification than in degenerative disease. A review by Yang et al. showed a pooled AUC of 0.95 in 14 studies on fractures. Our results outperforms almost all networks classing OA that we have found, with the notable exception of a paper by Tiulpin et al. [14].

Our study has some limitations. We were unable to collect a large enough test set, leading to the necessity of using the validation set evaluate for outcome measures. We tried to counteract this by putting the validation set through double validation to increase its accuracy. Using a test set that the network has not been exposed to, preferably classed by experts, would lead to more accurate assessment of true network performance. We also did not have an external dataset for validation. Furthermore, we were unable to obtain a consistent proportion between the training and validation set.

During the labelling phase we noted that the number of cases in some Hamada grades was rather low. These groups were attempted to be bolstered by more specific selection, with limited success. Radiologists do not consistently comment on CTA-like changes, meaning our method of selection, querying the radiologist reports, could only extract a portion of probable CTA cases in our archive.

Future studies should incorporate a larger dataset for validation to improve the reliability of glenoid erosion assessment. Including a second dataset or performing external validation would also be beneficial. Additionally, alternative methods for selecting radiographs may enhance analysis in these rare cases, as radiologist reports often lack descriptions of glenoid erosion. Leveraging clinical information through natural language processing could further enhance the accuracy of early Hamada stage assessment, particularly when radiological data alone is insufficient. Studies also need to be performed on clinical implementation to see how AI-systems impact radiograph reviewers’ performance when used as guidance. One study showed AI-assisted analysis by emergency medicine physicians reducing relative error rate in fracture detection by 47% when compared with unaided analysis [27]. Further clinical studies of this type are required to prove the safety and efficacy of AI-assisted analysis in medical imaging.

## Conclusions

To conclude, this study is the first of its kind in applying an ANN to classification of CTA and glenoid erosion on plain shoulder radiographs. The network performed excellently in recognizing more severe cases and showed adequate results on earlier stages. The potential clinical implications of our findings are substantial. AI systems could offer standardized CTA classification, facilitating connections between radiological findings and treatment outcomes. Moreover, in settings with limited radiologist availability, AI assistance could prove transformative, ensuring accurate and timely diagnoses, ultimately improving patient care and outcomes.

## Data Availability

No datasets were generated or analysed during the current study.

## Abbreviations

AI: Artificial intelligence
AHI: Acromiohumeral interval
ANN: Artificial neural network
AUC: Area under curve
AVN: Avascular necrosis
CNN: Convolutional neural network
CTA: Cuff tear arthropathy
GPU: Graphics processing unit
MRI: Magnetic resonance imaging
OA: Osteoarthritis
ROC: Receiver operator characteristics

## References

[1] Neer CS, Craig EV, Fukuda H. Cuff-tear arthropathy. J Bone Joint Surg Am. 1983;65(9):1232–44. 6654936

[2] Rugg CM, Gallo RA, Craig EV, Feeley BT. The pathogenesis and management of cuff tear arthropathy. J Shoulder Elb Surg. 2018;27(12):2271–83. 10.1016/j.jse.2018.07.020 30268586

[3] Visotsky JL, Basamania C, Seebauer L, Rockwood CA, Jensen KL. Cuff tear arthropathy: pathogenesis, classification, and algorithm for treatment. J Bone Joint Surg Am. 2004;86–A(Suppl 2):35–40. 15691107

[4] Brorson S. Cuff tear arthropathy in the nineteenth century: ‘chronic rheumatic arthritis’ with ‘partial luxation upwards’ of the humeral head. Int Orthop. 2019;43(10):2415–23. 31388708 10.1007/s00264-019-04380-4

[5] Chard MD, Hazleman R, Hazleman BL, King RH, Reiss BB. Shoulder disorders in the elderly: a community survey. Arthritis Rheum. 1991;34(6):766–9. 2053923 10.1002/art.1780340619

[6] Ozaki J, Fujimoto S, Nakagawa Y, Masuhara K, Tamai S. Tears of the rotator cuff of the shoulder associated with pathological changes in the acromion. A study in Cadavera. J Bone Joint Surg Am. 1988;70(8):1224–30. 3417708

[7] Milgrom C, Schaffler M, Gilbert S, van Holsbeeck M. Rotator-cuff changes in asymptomatic adults. The effect of age, hand dominance and gender. J Bone Joint Surg Br. 1995;77(2):296–8. 7706351

[8] Minagawa H, Yamamoto N, Abe H, Fukuda M, Seki N, Kikuchi K, et al. Prevalence of symptomatic and asymptomatic rotator cuff tears in the general population: from mass-screening in one village. J Orthop. 2013;10(1):8–12. 24403741 10.1016/j.jor.2013.01.008 PMC3768248

[9] Hamada K, Fukuda H, Mikasa M, Kobayashi Y. Roentgenographic findings in massive rotator cuff tears. A long-term observation. Clin Orthop Relat Res. 1990;254:92–6. 2323152

[10] Litjens G, Kooi T, Bejnordi BE, Setio AAA, Ciompi F, Ghafoorian M, et al. A survey on deep learning in medical image analysis. Med Image Anal. 2017;42:60–88. 28778026 10.1016/j.media.2017.07.005

[11] Chung SW, Han SS, Lee JW, Oh KS, Kim NR, Yoon JP, et al. Automated detection and classification of the proximal humerus fracture by using deep learning algorithm. Acta Orthop. 2018;89(4):468–73. 29577791 10.1080/17453674.2018.1453714 PMC6066766

[12] Sirveaux F, Favard L, Oudet D, Huquet D, Walch G, Molé D. Grammont inverted total shoulder arthroplasty in the treatment of glenohumeral osteoarthritis with massive rupture of the cuff. Results of a multicentre study of 80 shoulders. J Bone Joint Surg Br. 2004;86(3):388–95. 15125127 10.1302/0301-620x.86b3.14024

[13] von Schacky CE, Sohn JH, Liu F, Ozhinsky E, Jungmann PM, Nardo L, et al. Development and validation of a multitask deep learning model for severity grading of hip osteoarthritis features on radiographs. Radiology. 2020;295(1):136–45. 32013791 10.1148/radiol.2020190925 PMC7104703

[14] Tiulpin A, Saarakkala S. Automatic grading of individual knee osteoarthritis features in plain radiographs using deep convolutional neural networks. Diagnostics (Basel). 2020;10(11):932. 33182830 10.3390/diagnostics10110932 PMC7697270

[15] Elsharkawi M, Cakir B, Reichel H, Kappe T. Reliability of radiologic glenohumeral osteoarthritis classifications. J Shoulder Elb Surg. 2013;22(8):1063–7. 10.1016/j.jse.2012.11.007 23375877

[16] Hartzler RU, Steen BM, Hussey MM, Cusick MC, Cottrell BJ, Clark RE, et al. Reverse shoulder arthroplasty for massive rotator cuff tear: risk factors for poor functional improvement. J Shoulder Elb Surg. 2015;24(11):1698–706. 10.1016/j.jse.2015.04.015 26175311

[17] He K, Zhang X, Ren S, Sun J. Delving Deep into Rectifiers: Surpassing Human-Level Performance on ImageNet Classification. In 2015 [cited 2020 Feb 4]. pp. 1026–34. Available from: https://www.cv-foundation.org/openaccess/content_iccv_2015/html/He_Delving_Deep_into_ICCV_2015_paper.html

[18] Hajian-Tilaki K. Receiver operating characteristic (ROC) curve analysis for medical diagnostic test evaluation. Casp J Intern Med. 2013;4(2):627–35. PMC3755824 24009950

[19] Mandrekar JN. Simple statistical measures for diagnostic accuracy assessment. J Thorac Oncol. 2010;5(6):763–4. 20502268 10.1097/JTO.0b013e3181dab122

[20] Youden WJ. Index for rating diagnostic tests. Cancer. 1950;3(1):32–5. 15405679 10.1002/1097-0142(1950)3:1<32::aid-cncr2820030106>3.0.co;2-3

[21] Thomas KA, Kidziński Ł, Halilaj E, Fleming SL, Venkataraman GR, Oei EHG, et al. Automated classification of radiographic knee osteoarthritis severity using deep neural networks. Radiol Artif Intell. 2020;2(2):e190065. 32280948 10.1148/ryai.2020190065 PMC7104788

[22] Fehringer EV, Rosipal CE, Rhodes DA, Lauder AJ, Puumala SE, Feschuk CA, et al. The radiographic acromiohumeral interval is affected by arm and radiographic beam position. Skeletal Radiol. 2008;37(6):535–9. 18343920 10.1007/s00256-008-0467-y

[23] Kopka M, Fourman M, Soni A, Cordle AC, Lin A. Can glenoid wear be accurately assessed using x-ray imaging? Evaluating agreement of x-ray and magnetic resonance imaging (MRI) Walch classification. J Shoulder Elb Surg. 2017;26(9):1527–32. 10.1016/j.jse.2017.03.014 28483433

[24] Fraenkel L, Lavalley M, Felson D. The use of radiographs to evaluate shoulder pain in the ED. Am J Emerg Med. 1998;16(6):560–3. 9786537 10.1016/s0735-6757(98)90218-2

[25] Frénay B, Kaban A. A Comprehensive Introduction to Label Noise. In 2014 [cited 2023 Oct 15]. Available from: https://dial.uclouvain.be/pr/boreal/object/boreal:156597

[26] Sheehy L, Culham E, McLean L, Niu J, Lynch J, Segal NA, et al. Validity and sensitivity to change of three scales for the radiographic assessment of knee osteoarthritis using images from the multicenter osteoarthritis study (MOST). Osteoarthritis Cartilage. 2015;23(9):1491–8. 26003948 10.1016/j.joca.2015.05.003 PMC4831715

[27] Lindsey R, Daluiski A, Chopra S, Lachapelle A, Mozer M, Sicular S, et al. Deep neural network improves fracture detection by clinicians. Proc Natl Acad Sci U S A. 2018;115(45):11591–6. 30348771 10.1073/pnas.1806905115 PMC6233134

